# Effect of High Pressure Homogenization-Modified Soy 11S Globulin on the Gel and Rheological Properties of Pork Myofibrillar Protein

**DOI:** 10.3390/foods12040810

**Published:** 2023-02-14

**Authors:** Qingfeng Ge, Yuehao Wu, Na Yuan, Zhaoyang Jia, Rui Liu, Fei Lu, Hanjun Ma, Zhuangli Kang

**Affiliations:** 1 School of Food Science and Technology, Yangzhou University, Yangzhou 225127, China; 2 School of Food Science, Henan Institute of Science and Technology, Xinxiang 453003, China; 3 School of Tourism and Cuisine, Industrial Engineering Center for Huaiyang Cuisin of Jiangsu Province, Yangzhou University, Yangzhou 225127, China

**Keywords:** soy 11S globulin, myofibrillar protein, texture, rheological property, microstructure

## Abstract

The changes in texture and rheological characteristics, water holding capacity, and microstructure of pork myofibrillar protein with high-pressure homogenization-modified (0–150 MPa) soy 11S globulin were studied. The cooking yield, whiteness values, texture properties, shear stress, initial apparent viscosity, storage modulus (G′), and loss modulus (G″) of pork myofibrillar protein with high-pressure homogenization-modified soy 11S globulin were significantly increased (*p* < 0.05) compared with the sample of 0 MPa, and centrifugal yield was significantly decreased, except for the sample of 150 MPa. Therein, the sample of 100 MPa had the largest values. Meanwhile, the water and proteins bonded more tightly because the initial relaxation times of T_2b_, T_21_ and T_22_ from pork myofibrillar protein with high-pressure homogenization-modified soy 11S globulin were shorter (*p* < 0.05). Overall, the water-holding capacity, gel texture and structure, and rheological properties of pork myofibrillar protein could improve when adding soy 11S globulin treated with 100 MPa.

## 1. Introduction

Myofibrillar protein has excellent gelling ability and plays an important role in the processing of meat products [1,2]. It forms a stable gel network structure through heat denaturation, which gives meat products good texture, taste, and flavour. To reduce the nutrient loss of meat products during the cooking process, soy protein isolate is added as a binder to meat products to not only improve their quality and yield but also reduce production costs [3,4]. Soy protein isolate is a plant protein that can enhance health activity and is effective in preventing cardiovascular diseases [5,6]. Part of the functional properties of soy protein isolate can simulate the role of fat in the processing of meat products and can be used as a fat substitute to produce low-fat products, which meet current people’s requirements for healthy food. Soy protein isolate usually needs to be processed to better interact with myofibrillar protein and have a beneficial effect. However, there is no interaction between natural soy protein and meat protein [7].

Soy proteins mainly comprise 7S and 11S globulins, which comprise over 70% of the total soy protein content [8]. Soy 7S and 11S globulins will not expand until the heating temperature reaches 77 °C and 92 °C, respectively, exposing the reaction group [9,10]. On the other hand, the complete denaturation temperature of myosin (approximately 67 °C) is lower than that of soy 7S and 11S globulins [11,12]. However, the conformation of myosin changes from 30 °C to 50 °C, with pyrolyzing away from non-covalent bonds and exposing the protein groups and then forming the aggregation of myosin heads [13]. Thus, it is a difficult question to address how the interaction between soy 11S globulin and meat protein can be improved.

At present, the technology of high-pressure homogenization has been used in the food industry to improve the quality and affect the enzyme activity and the conformation of the protein, in addition to killing bacteria and other microorganisms using high-speed impact, high-frequency vibration and cavitation, which are generated during the processing [14,15]. Previous studies have reported that high-pressure homogenization can directly affect the secondary structure of soy protein to improve its functional properties [16,17]. Xu, Mukherjee, and Chang [12] found that the use of high-pressure homogenization (70, 140, and 210 MPa) can improve the functional properties of soy protein isolate, 7S globulin and 11S globulin. Our previous studies have shown that high-pressure homogenization (50–150 MPa) improves the physicochemical, foaming, and gel properties of 11S globulin by increasing the magnitude of protein zeta potential, solubility, surface hydrophobicity, and free sulfhydryl groups, but excessive pressure treatment at 150 MPa caused solubility, surface hydrophobicity, exposure to free sulfhydryl, and elasticity index to decrease. Thus, the pressure of 100 MPa but not 150 MPa mostly affects the properties of the soy 11S globulin systems [18]. However, as far as we know, few studies have investigated the use of high-pressure homogenization-modified soy 11S globulin to improve the gel and rheological properties of pork myofibrillar protein. Therefore, the aim of this study was to investigate the use of high-pressure homogenization-modified (50 MPa, 100 MPa, and 150 MPa) soy 11S globulin to improve the water holding capacity, color, texture properties, rheology characteristics, and microstructure of pork myofibrillar protein.

## 2. Materials and Methods

### 2.1. Raw Materials and Ingredients

Chilled pork *longissimus lumborum* (pH, 5.75 ± 0.01) was supplied from 16 pigs (100 ± 5 kg, Xinxiang Gaojin Food Co. LTD., Xinxiang, China). Soybean (Shangdou-6; protein, 43.02 ± 0.31%; lipid, 19.42 ± 0.21%) was purchased from Shangqiu Academy of Agricultural Sciences, China. All the chemical reagents, such as HCl, Tris-HCl, K_2_HPO_4,_ and KH_2_PO_4_, were of analytical-grade purity.

### 2.2. Extraction of Pork Myofibrillar Protein

According to the method of Li, Zhang, Lu, and Kang [19], the myofibrillar protein was extracted from the pork *longissimus lumborum*. After removing the visible-fat and connective tissue, the meat was cut into small pieces and homogenized in four volumes of buffer (100 mmol/L Tris, 10 mmol/L EDTA, pH 8.3) by a homogenizer (T25, IKA, Staufen, Germany). The homogenates were centrifuged (4 °C) at 1000× *g* for 20 min (Sorvall LYNX4000, Thermo Fisher Scientific, Langenselbold, Germany). After decanting the supernatant, the precipitate was dispersed in four volumes of buffer (100 mmol/L KCl, 20 mmol/L K_2_HPO_4_/KH_2_PO_4_, 2 mmol/L MgCl_2_, 1 mmol/L EGTA, 1 mmol/L NaN_3_, pH 7.0) and centrifuged at 1000× *g* for 10 min under the same conditions described above for another two times. After that, the pellet was resuspended in four volumes of buffer (100 mmol/L KCl, 20 mmol/L K_2_HPO_4_/KH_2_PO_4_, 2 mmol/L MgCl_2_, 1 mmol/L EGTA, 1 mmol/L NaN_3_, 1% Triton X-100, pH 7.0) and then centrifuged (1500× *g* for 10 min) under the same conditions described above once more. After decanting the supernatant, pellets were resuspended in four volumes of a 0.1 mol/L KCl solution and centrifuged at 1500× *g* for 10 min under the same conditions mentioned above one more time. After that, the pellet was resuspended in four volumes of 0.1 mol/L NaCl solution, centrifuged at 1500× *g* for 10 min under the same conditions described above, and repeated once again. Finally, the purified myofibrillar protein solution was obtained, stored at 4 °C, and used within 24 h.

### 2.3. Extraction of Soy 11S Globulin and HPH Treatment

According to the method of our previous study [18], soy 11S globulin was extracted from soybean. Briefly, soy was powdered and oil-removed to obtain defatted soy powder. Then, the powder was mixed with 15-fold (*w*/*v*) of 0.03 M Tris-HCl buffer (pH, 8.50) by dipping it at 45 °C for 1 h, followed by centrifugation at 8000× *g* for 30 min to remove the precipitation, and the pH of the supernatant was adjusted to 6.4 with 2 M HCl. After overnight storage at 4 °C, the precipitate was collected by centrifugation (6000× *g*, 20 min, 4 °C) and frozen using a vacuum freeze dryer (Alpha 1–2 LD plus, Christ, Osterode, Germany) at −60 °C for 24 h. The protein content of the soy 11S globulin solution was determined by the micro-Kjeldahl method. After that, approximately 10 g of soy 11S globulin and 90 g of deionized water (10 °C) were mixed in uniformity using a glass rod, and the solution was homogenized twice (0, 50, 100, and 150 MPa) using a high-pressure nano-homogenizer (FGP 12805, Stansted Fluid Power Ltd., Gloucestershire, UK), respectively.

### 2.4. Preparation of Pork Myofibrillar Protein and Soy 11S Globulin Mixture Solution

The myofibrillar protein was adjusted to 60 mg/mL with a phosphate buffer solution of 50 mmol/L (K_2_HPO_4_/KH_2_PO_4_, pH 6.0). After that, 80 g myofibrillar protein solution, 20 g soy 11S globulin solution, and 2 g NaCl were mixed uniformly using a homogenizer (T25, IKA, Germany) at 3000 rpm for 50 s in an ice bath. The solution was then centrifuged at 500× *g* (4 °C, 3 min) to remove any air bubbles, then 10 g mixed solution was poured into a beaker and heated in an 80 °C water bath for 20 min.

### 2.5. Cooking Yield

After being stored overnight at 2 ± 2 °C, the cooked protein solution was taken out of the beaker and weighed. The cooking yield was calculated using the following formula:

Cooking yield (%) = Weight of proteins solution after cooking/Weight of proteins solution before cooking × 100%.(1)

### 2.6. Centrifugal Yield

Approximately 10 g cooked protein solution was transferred to a centrifuge tube and centrifuged at 5000× *g* for 10 min at 4 °C using a centrifuge (LYNX4000, Thermo Fisher Scientific, Pittsburgh, PA, USA), then the water from the centrifuge was weighed. The calculation formula was as follows:

Centrifugal loss (%) = 1 − Weight of the water from the centrifuge/Weight of proteins solution after cooking × 100%(2)

### 2.7. Color

The L*, a*, and b* values of the cooked protein solution were measured by a colorimeter (CR-400, Minolta, Tokyo, Japan) with an aperture of 8 mm, a 10° observer angle, and a D65 illuminant and calibrated against a white plate (L* = 94.56, a* = −0.18, b* = 1.62). The whiteness value calculation formula was as follows [20]:

Whiteness value = 100 − [(100 − L*)^2^ + a*^2^ + b*^2^]^1/2^ (3)

### 2.8. Texture Profile Analysis

The texture properties were determined according to the method of Zhu et al. [21] using a texture analyzer (TA-XT.plus, Stable Micro System Ltd., Godalming, UK) fitted with a cylinder probe (P/36R). The cooked protein solution was cut into a cylinder (diameter, 10 mm; height, 15 mm). Test parameters were as follows: pre-text speed 2.0 mm/s; text speed 2.0 mm/s; post-text speed 5.0 mm/s; strain 50%, time 5.0 s; trigger type, auto; and trigger force 5 g for TPA measurement. The samples were cut in two to obtain 20 mm depth and 25 mm diameter strips. Finally, hardness, springiness, cohesiveness, and resilience were obtained.

### 2.9. Rheological Properties Measurement

Shear stress and apparent viscosity of the protein solution were measured with a dynamic rheometer (HAAKE MARS, Thermo Fisher Scientific, Pittsburgh, PA, USA) equipped with the P60 probe with a gap of 1 mm, and the protein solution was evenly applied to the plate and preserved for 5 min before assays. The parameters were set as follows: the shear rate, temperature, and time were 10 s^−1^~400 s^−1^, 25 °C, and 330 s, respectively.

The dynamic rheological properties of proteins solution were measured using a dynamic rheometer (HAAKE MARS, Thermo Fisher Scientific, Pittsburgh, USA) according to the method of Kang et al. [22]. A 50 mm parallel steel plate geometry with a 0.5 mm gap was used. The raw protein solution was placed between the flat parallel plates with its perimeter coated with a thin layer of silicone oil to prevent dehydration. The sample was heated at a rate of 2 °C/min and continuously sheared in an oscillatory mode at a fixed frequency of 0.1 Hz. The storage modulus (G′) and loss modulus(G″) were recorded continuously from 20 °C to 80 °C.

### 2.10. Low Field Nuclear Magnetic Resonance (LF-NMR)

According to the method of Li, Kang, Sukmanov, and Ma [11], approximately 2 g of the cooked protein solution was placed in a 15 mm glass tube and inserted in the NMR probe of a Pulsed NMR analyzer (NMI20-040 V-1, Niumag Electric Corporation, Shanghai, China) operated at a resonance frequency of 22 MHz at 32 °C. The parameters used were as follows: η~value (the time between 90° pulse and 180° pulse) was 200 μs; 32 repeated scans with a time interval of 110 ms; and 12,000 echoes.

### 2.11. Scanning Electron Microscopy

The microstructure of the cooked protein solution was determined using scanning electron microscopy (Hitachi-S-3000N, Hitachi High Technologies Corp., Toyoko, Japan) according to the procedure reported by Haga and Ohashi [23]. Briefly, cubic samples (3 × 3 × 3 mm^3^) obtained from cooked protein solution were fixed for 24 h at 4 °C in 0.1 M phosphate buffer (pH 7.0) containing 2.5% glutaraldehyde and washed in 0.1 M phosphate buffer (pH 7.0) for 10 min. Then, the samples were post-fixed for 5 h in the same buffer with 1% osmium tetroxide. The post-fixed samples were washed three times with 0.1 M phosphate buffer (pH 7.0) for 10 min and dehydrated in incremental concentrations of ethanol (50, 60, 70, 80, 90, and 95%, and three times with 100%) for 10 min for each solution.

### 2.12. Statistical Analysis

The experiment was repeated four times on different occasions (*n* = 4) using different samples and recorded as mean values and standard deviation (mean ± SD). The data were analyzed through the general linear model (GLM) procedure using the statistical software package SPSS v.18.0, considering the treatments (different homogenization pressure levels) as a fixed effect and the replicates as a random effect. Significant differences between means were identified by the LSD procedure. The difference between means was considered significant at *p* < 0.05.

## 3. Result and Discussion

### 3.1. Cooking Yield

The changes in the cooking yield of pork myofibrillar protein solution with high-pressure homogenization-modified soy 11S globulin (PH11S) are shown in Table 1. Compared with the sample of 0 MPa, the cooking yield of PH11S was increased significantly (*p* < 0.05), and the highest cooking yield was observed at 100 MPa; meanwhile, the 50 MPa and 150 MPa were not significantly different (*p* > 0.05). The possible reason is that the structures of soy 11S globulin were broken during the homogenization treatment. For example, the particle size and turbidity were decreased, which led to the emulsification properties and a decrease in water-holding capacity [18]. In addition, the soy 11S globulin solution treated by high-pressure homogenization could combine with the myofibrillar protein and then form a stable gel network during heating; therefore, the gel binds more water and improves water retention [24]. On the other hand, due to the excessive expansion of soy 11S globulin and the re-polymerization of the subunit structure dissociated under 150 MPa, its capacity of holding water was decreased, thus the cooking yield of myofibrillar protein gel was decreased [25,26].

### 3.2. Centrifugal Yield

The protein gel forms a dense network structure that can confine water. The more stable the gel structure, the stronger the water confinement ability. Water-holding capacity represents the ability of a protein to bind water and is an important property of meat product quality assessment [27]. Table 1 shows the effect of high-pressure homogenization-modified soy 11S globulin on the centrifugal yield of cooked myofibrillar protein solution. Compared with the sample of 0 MPa, the centrifugal yield of PH11S was increased significantly (*p* < 0.05), and the highest centrifugal yield was observed at 100 MPa; meanwhile, the 50 MPa and 150 MPa were not significantly different (*p* > 0.05). The result was in agreement with the result of the cooking yield (Table 1). It is possible that high-pressure homogenization treatment increased the surface hydrophobicity and free sulfhydryl groups of soy 11S globulin, leading to improved surface tension, initial apparent viscosity, and shear stress and enhancing the water-holding capacity [18]. On the other hand, due to more soy 11S globulins being dissolved and more surface hydrophobicity and free sulfhydryl groups being exposed after high-pressure homogenization treatment, the interaction between soy 11S globulin and meat protein can play a certain role in modification [28]. Therein, excessive pressures over-swell the protein and expose part of the hydrophobic residues, enhancing the hydrophobic effect of the protein, and thereby reducing the ability to bind water [18].

### 3.3. Color

The color of the gel can also be used as an important indicator of its quality. The effect of high-pressure homogenization-modified soy 11S globulin on the color of cooked myofibrillar protein solution is shown in Table 1. Compared with the sample of 0 MPa, the L*, a*, b*, and whiteness values of cooked PH11S were significantly increased (*p* < 0.05), except for the sample of 50 MPa. The reason is that the cooked PH11S had higher water content than the sample of 0 MPa (Table 1), causing the increase in the L*, a*, b*, and whiteness values. Meanwhile, after high-pressure homogenization treatment, the whiteness value of the cooked pork myofibrillar protein solution was increased, indicating that protein denaturation or pigment oxidation occurred during the gel formation process [29]. Hwang et al. [30] also showed that the color difference of tilapia muscle protein gel is related to the degree of protein denaturation.

### 3.4. Texture Properties

The effect of high-pressure homogenization-modified soy 11S globulin on the texture properties of cooked myofibrillar protein solution is shown in Table 2. Compared with the sample of 0 MPa, the hardness, springiness, cohesiveness, and resilience of cooked myofibrillar protein solution with high-pressure homogenization-modified soy 11S globulin were significantly increased (*p* < 0.05), and the sample of 100 MPa has the highest hardness, cohesiveness, and resilience, except springiness. Natural soy 11S globulin is difficult to cross-link with myofibrillar protein and cannot enhance the stability of the protein gel, but it reduces the gel strength of the protein [10,31]. Therein, because the solubility and Zeta potential of soy 11S globulin were significantly increased after high-pressure homogenization treatment, leading to more surface hydrophobicity and free sulfhydryl groups being exposed, caused more binding sites of soy 11S globulin being formed, which resulted in more interactions between soy 11S globulin and myofibrillar protein during heating [18,32].

### 3.5. Rheological Analysis

#### 3.5.1. Shear Stress and Apparent Viscosity

**Figure 1:**
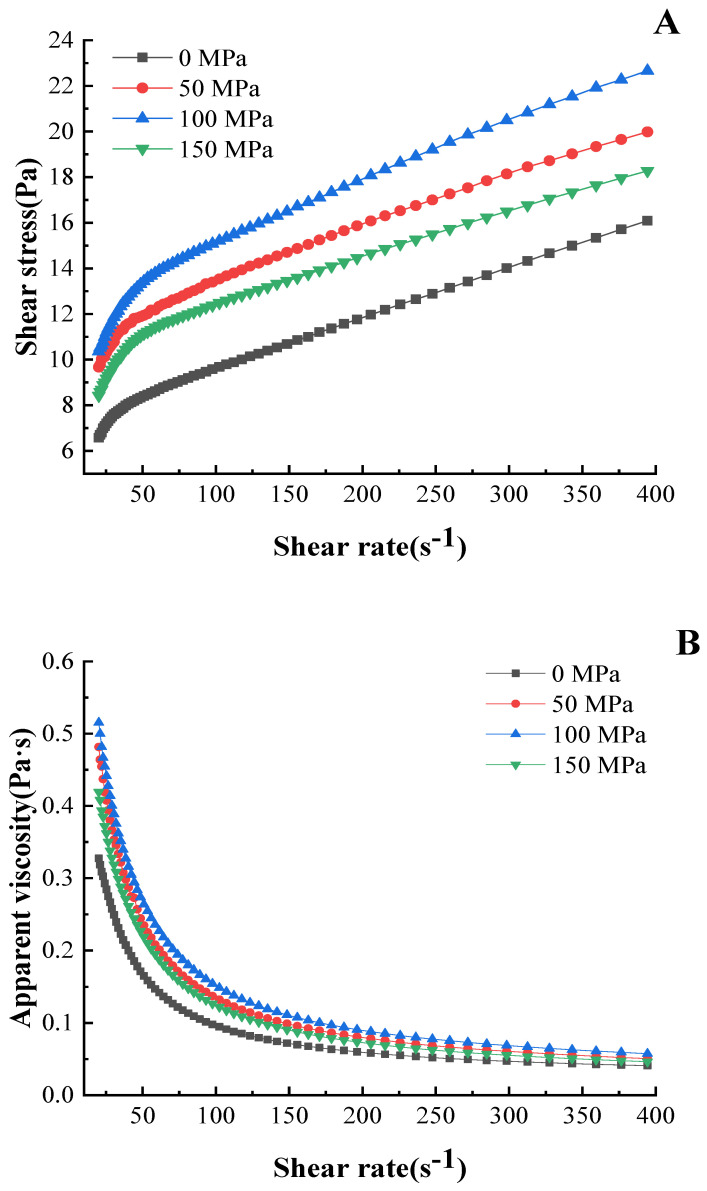
The shear stress (Pa) and apparent viscosity (Pa•s) of pork myofibrillar protein with high-pressure homogenization-modified soy 11S globulin. (**A**): shear stress (Pa); (**B**): apparent viscosity (Pa•s).

The rheological properties can describe the speed and resistance of the protein solution under the action of external force [33,34]. Figure 1 shows the changes in the shear stress and apparent viscosity of pork myofibrillar protein with high-pressure homogenization-modified soy 11S globulin. It can be seen from Figure 1A that the shear stress of pork myofibrillar protein solution was significantly increased with the increase in shear rate. Meanwhile, compared with the sample of 0 MPa, the shear stress was significantly increased with the increase in homogenization pressures, and the sample of 100 MPa has the highest shear stress at the same shear rate.

The apparent viscosity of pork myofibrillar protein with high-pressure homogenization-modified soy 11S globulin is shown in Figure 1B. Due to the apparent viscosity showing a downward trend with the increase in shear rate, the myofibrillar protein solution was a pseudoplastic fluid [35]. Compared with the sample of 0 MPa (0.69 Pa•s), the initial viscosity of the myofibrillar protein solution with high-pressure homogenization-modified soy 11S globulin significantly increased, and the sample of 100 MPa has the highest initial viscosity (1.18 Pa•s). This was possibly due to the changes in the physical and chemical properties of soy 11S globulin after the high pressure homogenization treatment. Some hydrophobic and free sulfhydryl groups inside the molecule were exposed due to the loose structure [18], which enhanced the combination between soy 11S globulin and myofibrillar protein and led to the increase in the asymmetry of the molecule; therefore, the viscosity increased, and the diffusion coefficient decreased.

#### 3.5.2. G′ and G″

**Figure 2:**
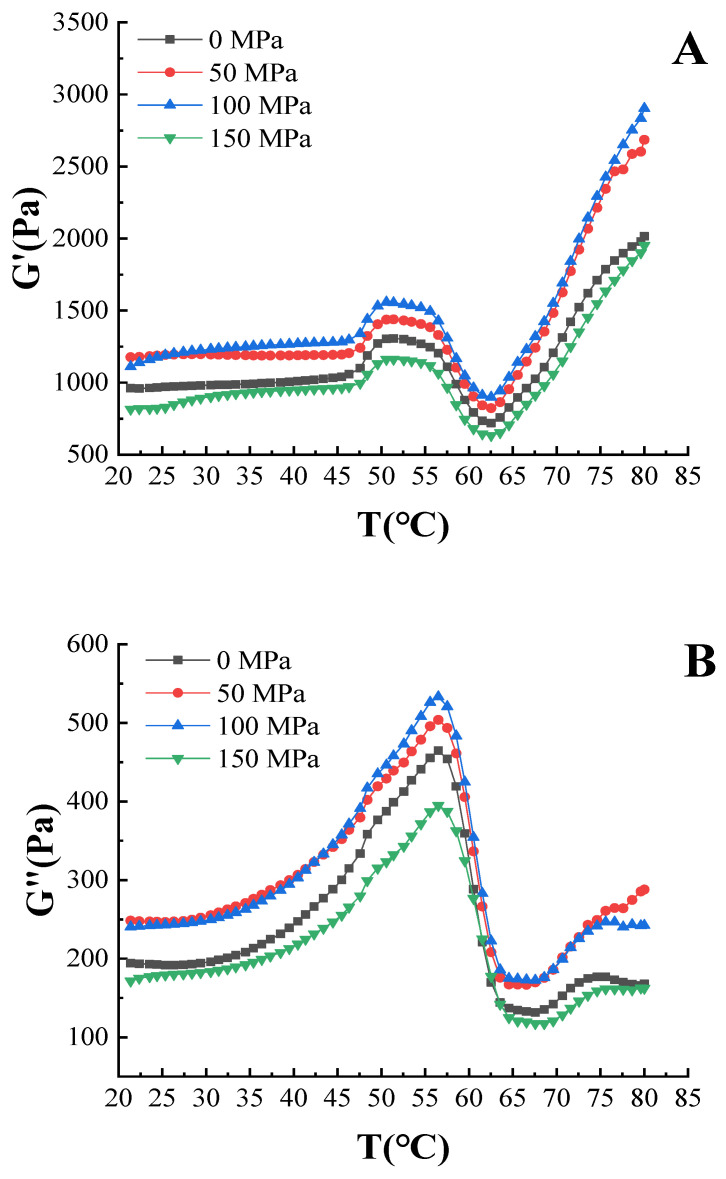
The storage modulus(G′) and loss modulus(G″) of pork myofibrillar protein with high-pressure homogenization-modified soy 11S globulin. (**A**): storage modulus(G′); (**B**): loss modulus(G″).

The effect of high-pressure homogenization-modified soy 11S globulin on the G′ and G″ of myofibrillar protein solution is shown in Figure 2. The G′ represents the energy stored by the protein gel due to elastic deformation during the deformation process and can reflect the strength of the gel, while the G″ explains the viscous behavior of the liquid-like components [21,36]. It can be seen from Figure 2A that all the samples had similar changing trends with three stages. When the temperature was increased to approximately 45 °C, the first peak of a wave was generated and formed a peak at approximately 51 °C. The general explanation at this stage is that the degeneration of the myosin head leads to an increase in G′ value and the initial formation of a gel network [37]. Next, the G′ value showed a downward trend from approximately 51 °C to 61 °C, as the tail of myosin was unfolded with the increase in temperature, which increased the fluidity of the gel system [38]. Thereafter, due to myosin being completely denatured and unfolded to form a random coil structure, which leads to an increase in the degree of cross-linking of the protein structure and the formation of an irreversible gel, the G′ value sharply increased from approximately 61 °C to 80 °C [39,40]. Compared with the sample of 0 MPa, the G′ values of the sample of 50 MPa and 100 MPa were higher during heating. This is because the high-pressure homogenization treatment exposed more amino acid residue sites inside the soy 11S globulin molecule and improved the hydration properties of the protein particles [18], which caused better cross-linking with myofibrillar protein and improved the stability of the sol and gel structure.

The changing trend of G″ was basically the same as the G′ with three stages (Figure 2B). A rapid increase in G″ of myofibrillar protein solution was observed from approximately 20 °C to 55 °C, and the result indicated that the increase of viscous component of myofibrillar protein solution, which was caused by actin denaturation and protein cross-linking. Next, because the denaturation of myosin is less viscous, a sharp decline in G″ was observed from approximately 55 °C to 63 °C. Next, a slight increase was observed from approximately 63 °C to 80 °C. Compared with the sample of 0 MPa, the G″ values of the sample of 50 MPa and 100 MPa were higher during heating, indicating that pork myofibrillar protein with high-pressure homogenization-modified (50 MPa and 100 MPa) soy 11S globulin exhibited a better elastic and viscous behavior. Therein, the G″ values were smaller than the G′ values during heating, which implied that the myofibrillar protein solution has an elastic property rather than a viscous property.

### 3.6. LF-NMR

**Figure 3:**
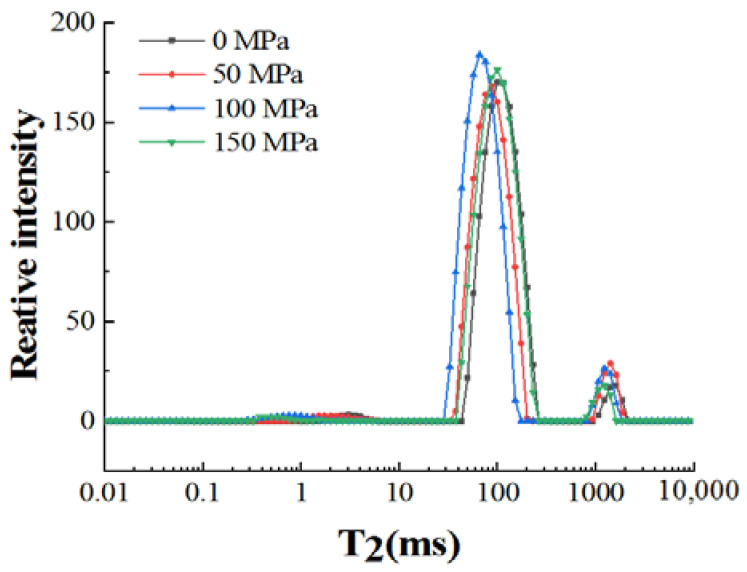
The changes of LF-NMR relaxation time of cooked pork myofibrillar protein with high-pressure homogenization-modified soy 11S globulin.

LF-NMR can non-destructively detect the movement and distribution of water molecules in samples and is widely used in gelled meat products [41]. There is a correlation between the transverse relaxation time (T_2_) and the water-holding capacity of meat and meat products. The smaller the T_2_, the tighter the binding of water molecules and proteins [20,42]. Figure 3 and Table 3 show the changes in the initial relaxation time and peak ratio of cooked pork myofibrillar protein with high-pressure homogenization-modified soy 11S globulin. Three peaks from 0.01 m s to 10,000 m s are shown in Figure 3, and they are T_2b_, T_21,_ and T_22_, respectively. Therein, T_2b_, T_21,_ and T_22_ represent the bound water, immobilized water, and free water, respectively.

The initial relaxation times of T_2b_, T_21,_ and T_22_ from pork myofibrillar protein with high-pressure homogenization-modified soy 11S globulin were smaller (*p* < 0.05) than the samples of 0 MPa (Table 3), and the initial relaxation times of T_21_ from the samples of 150 MPa was the largest. The reason for this is that adding soy 11S globulin treated with 50 MPa and 100 MPa could form a better gel structure and improve water retention (Table 1 and Table 2). On the other hand, all the peak ratios of P_2b_ were not significantly different (*p* > 0.05). The sample of 50 MPa, 100 MPa, and 150 MPa had the largest peak ratio of P_21_, and the sample of 100 MPa and 150 MPa had the smallest peak ratio of P_22_. The reason is possible that the addition of soy 11S globulin treated over 50 MPa could enhance the protein cross-link between soy 11S globulin and myofibrillar protein, causing the fluidity of water to decline and leading to the immobilized water being increased and free water being decreased [43].

### 3.7. Microstructure

**Figure 4:**
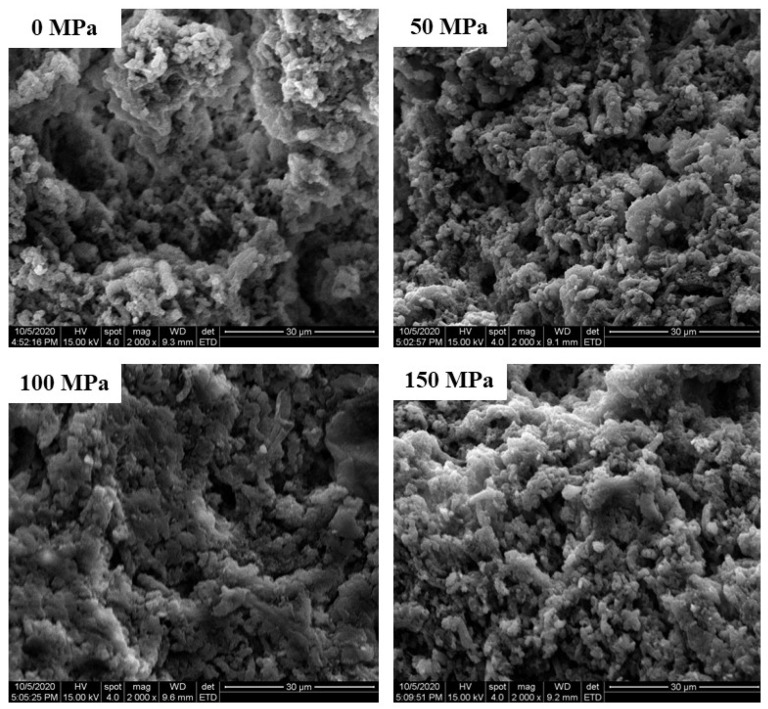
The microstructure of cooked pork myofibrillar protein with high-pressure homogenization-modified soy 11S globulin.

The microstructure of cooked pork myofibrillar protein with high-pressure homogenization-modified soy 11S globulin is shown in Figure 4. All the samples have similar microstructures with some cavities. The surface in the sample of 0 MPa and 150 MPa was rough and had some large aggregates formed by myofibrillar proteins, leading to the degree of low cross-linking between myofibrillar protein and soy 11S globulin [44,45]. Compared with the sample of 0 MPa, the void spaces of cooked myofibrillar protein in the sample of 50 MPa and 100 MPa were smaller and became smoother. It is possible that the soy 11S globulin treated with 50 MPa and 100 MPa had the highest solubility, the absolute value of Zeta potential, surface hydrophobicity, free sulfhydryl content, and disulfide bonds, as well as the gel properties [18]. The result was in agreement with the results of water-holding capacity, texture, and rheological properties (Table 1 and Table 2, and Figure 1 and Figure 2).

## 4. Conclusions

The result found that the addition of high-pressure homogenization-modified soy 11S globulin significantly affected the gel and rheological properties, and microstructure of pork myofibrillar protein. The addition of high-pressure homogenization-modified soy 11S globulin (50–150 MPa) significantly increased the cooking yield, L*, a*, b* and whiteness values, hardness, springiness, cohesiveness, and resilience of pork myofibrillar protein compared with the sample of 0 MPa, while the sample of 100 MPa had the highest values, except springiness. Because the sample of 100 MPa had the largest shear stress, initial apparent viscosity, G′ and G″, a uniform structure with good water holding capacity was formed. Thus, the addition of soy 11S globulin treated with 100 MPa could improve the water-holding capacity, gel, and rheological properties of pork myofibrillar protein.

## Figures and Tables

**Table 1:** The cooking yield (%), centrifugal yield (%), and color (L*, a* and b*, whiteness values) of pork myofibrillar protein with high-pressure homogenization-modified soy 11S globulin.

Homogenization Pressure	Cooking Yield (%)	Centrifugal Yield (%)	L* Value	a* Value	b* Value	Whiteness
0 MPa	62.93 ± 1.21 ^c^	64.97 ± 0.53 ^c^	84.34 ± 0.65 ^b^	−1.62 ± 0.03 ^b^	4.50 ± 0.10 ^c^	83.62 ± 0.61 ^b^
50 MPa	64.37 ± 0.61 ^b^	70.65 ± 1.11 ^b^	85.28 ± 1.14 ^b^	−1.56 ± 0.03 ^a^	5.51 ± 0.72 ^b^	84.20 ± 1.19 ^b^
100 MPa	67.38 ± 0.54 ^a^	74.37 ± 1.45 ^a^	87.40 ± 1.42 ^a^	−1.54 ± 0.05 ^a^	6.07 ± 0.26 ^ab^	85.91 ± 1.24 ^ab^
150 MPa	64.28 ± 0.85 ^b^	70.31 ± 1.01 ^b^	87.62 ± 0.63 ^a^	−1.52 ± 0.03 ^a^	6.28 ± 0.32 ^a^	86.02 ± 0.47 ^a^

Note: Each value represents the mean ± SD, *n* = 4. ^a–c^ Different parameter superscripts indicate significant differences (*p* < 0.05).

**Table 2:** The hardness, springiness, cohesiveness, and resilience of pork myofibrillar protein with high-pressure homogenization-modified soy 11S globulin.

Homogenization Pressure	Hardness (g)	Springiness	Cohesiveness	Resilience
0 MPa	225.31 ± 12.91 ^c^	0.879 ± 0.036 ^c^	0.491 ± 0.037 ^b^	0.206 ± 0.024 ^b^
50 MPa	308.14 ± 26.39 ^b^	0.917 ± 0.027 ^b^	0.576 ± 0.051 ^a^	0.265 ± 0.040 ^a^
100 MPa	344.95 ± 13.47 ^a^	0.933 ± 0.028 ^ab^	0.605 ± 0.027 ^a^	0.294 ± 0.031 ^a^
150 MPa	298.27 ± 15.86 ^b^	0.951 ± 0.006 ^a^	0.577 ± 0.029 ^a^	0.285 ± 0.016 ^a^

Note: Each value represents the mean ± SD, *n* = 4. ^a–c^ Different parameter superscripts indicate significant differences (*p* < 0.05).

**Table 3:** The initial relaxation time (ms) and peak ratio (%) of cooked pork myofibrillar protein with high-pressure homogenization-modified soy 11S globulin.

Homogenization Pressure	Initial Relaxation Time			The Peak Ratio		
	T_2b_	T_21_	T_22_	P_2b_	P_21_	P_22_
0 MPa	1.74 ± 0.69 ^a^	49.77 ± 5.68 ^b^	1072.27 ± 54.05 ^a^	1.76 ± 0.06 ^a^	95.66 ± 1.28 ^b^	3.12 ± 1.37 ^a^
50 MPa	1.04 ± 0.04 ^b^	37.64 ± 1.23 ^c^	932.61 ± 72.58 ^b^	1.72 ± 0.13 ^a^	97.87 ± 0.36 ^a^	0.80 ± 0.07 ^b^
100 MPa	0.28 ± 0.02 ^c^	32.74 ± 2.12 ^c^	811.13 ± 22.03 ^c^	1.69 ± 0.26 ^a^	97.54 ± 0.41 ^a^	0.29 ± 0.06 ^c^
150 MPa	0.37 ± 0.01 ^c^	73.28 ± 3.12 ^a^	811.13 ± 83.15 ^c^	1.81 ± 0.47 ^a^	98.07 ± 0.43 ^a^	0.28 ± 0.02 ^c^

Note: T_2b_, the initial relaxation time of bound water; T_21_, the initial relaxation time of immobilized water; T_22_, the initial relaxation time of free water; P_2b_, the peak ratio of bound water; P_21_, the peak ratio of immobilized water; P_22_, the peak ratio of free water. Each value represents the mean ± SD, *n* = 4. ^a–c^ Different parameter superscripts indicate significant differences (*p* < 0.05).

## Data Availability

Data is contained within the article.
